# Reconstituted High-Density Lipoprotein Modulates Activation of Human Leukocytes

**DOI:** 10.1371/journal.pone.0071235

**Published:** 2013-08-14

**Authors:** Rolf Spirig, Alexander Schaub, Alain Kropf, Sylvia Miescher, Martin O. Spycher, Robert Rieben

**Affiliations:** 1 Laboratory of Cardiovascular Research, Department of Clinical Research, University of Bern, Bern, Switzerland; 2 CSL Behring AG, Bern, Switzerland; Istituto Superiore di Sanità, Italy

## Abstract

An anti-inflammatory effect of reconstituted High Density Lipoprotein (rHDL) has been demonstrated in atherosclerosis and in sepsis models. An increase of adhesion molecules as well as tissue factor expression on endothelial cells in response to inflammatory or danger signals are attenuated by the treatment with rHDL. Here we show the inhibitory effect of rHDL on the activation of human leukocytes in a whole blood assay as well as on monocyte-derived human dendritic cells (DC). Multiplex analysis of human whole blood showed that phytohaemagglutinin (PHA)-induced secretion of the cytokines IL-1β, IL-1RA, IL-2R, IL-6, IL-7, IL-12(p40), IL-15 and IFN-α was inhibited. Furthermore, an inhibitory effect on the production of the chemokines CCL-2, CCL-4, CCL-5, CXCL-9 and CXCL-10 was observed. Activation of granulocytes and CD14^+^ monocytes by PHA is inhibited dose-dependently by rHDL shown as decreased up-regulation of ICAM-1 surface expression. In addition, we found a strong inhibitory effect of rHDL on toll-like receptor 2 (TLR2)- and TLR4-mediated maturation of DC. Treatment of DC with rHDL prevented the up-regulation of cell surface molecules CD80, CD83 and CD86 and it inhibited the TLR-driven activation of inflammatory transcription factor NF-κB. These findings suggest that rHDL prevents activation of crucial cellular players of cellular immunity and could therefore be a useful reagent to impede inflammation as well as the link between innate and adaptive immunity.

## Introduction

A beneficial effect of treatment with reconstituted High Density Lipoprotein (rHDL), containing plasma derived apolipoprotein A-I (apoA-I) and phosphatidylcholine (PC), was described in models for atherosclerosis, myocardial infarction, stroke and endotoxemia, and in clinical trials demonstrating effects on atherosclerotic plaques [1], [2]. Protective properties of rHDL on the endothelium have been described to be mediated by inhibiting up-regulation of inflammatory adhesion molecules like ICAM-1 (CD54), VCAM-1 (CD106) and E-selectin (CD62E) on endothelial cells (EC) [3] as well as reduced thrombin induced tissue-factor (TF) expression [4], and increasing bioavailability of NO [5]. A study in humans showed that rHDL reduces plasma levels of TNF-α and expression of CD11b on monocytes [2]. Protection against cardiac ischemia/reperfusion (I/R) injury was demonstrated by a reduced cardiac content of TNF-α and enhanced secretion of prostaglandin I_2_ and -E_2_ in a Langendorff perfusion model [6]. In myocardial infarction in rats, infusion of rHDL showed an increased phosphorylation of the MAP kinase family member extracellular-signal-related kinase (ERK) [7].

Physiologically HDL is involved in lipid homeostasis, especially in reversed cholesterol transport. Furthermore, HDL has been suggested to reduce atherosclerosis by suppression of hematopoietic stem cell proliferation, leukocytosis and monocytosis as another anti-atherogenic effect taking place prior the anti-inflammatory effect of this molecule [8]. A disturbed cholesterol efflux is associated with development of diseases such as atherosclerosis or acute coronary syndrome (ACS). Interestingly, cholesterol accumulation in cellular membranes has been shown to activate toll-like receptors (TLR) in macrophages [9]. Furthermore, endogenous TLR agonists are rapidly released under conditions of inflammation and tissue damage [10]–[13]. Recent studies have highlighted the involvement of TLR2 and TLR4 in the early inflammatory process of I/R injury *in vivo* [14], [15], atherosclerosis [16] or ACS [17].

rHDL can be considered a substance which attenuates the pro-inflammatory effects of many mediators of innate immunity. We hypothesized, therefore, that rHDL might influence inflammatory responses of important cellular players of innate immunity, namely neutrophils and monocytes, as well as professional antigen presenting cells (APC), which are crucially involved in linking the innate with the adaptive immune system.

Granulocytes and in particular neutrophils are the most abundant leukocytes in the human body and provide a crucial first line of defense against infections [18]. On the other hand, activation of neutrophils may be detrimental in situations of acute and chronic inflammation with release of proteolytic enzymes as well as oxygen free radicals increasing damage in diseases such as myocardial infarction [19] or in graft rejection [20]. Furthermore, neutrophils are a rich source of various proinflammatory cytokines and chemokines as e.g. CXCL-8 [21]. Monocytes are a second important cell type involved in inflammation. They have been described to have antigen-presenting activity [22] and to release a whole set of pro- and anti-inflammatory cytokines, thus being major contributors to cellular inflammation in diseases such as I/R injury [23]–[25]. In addition, monocytes can differentiate into dendritic cells (DC) which are very potent APC and pivotal for the initiation of T-cell mediated immune responses, as seen for example in allograft rejection as well as in tolerance induction [26]. In addition, DC are producers of various cytokines and chemokines and a major source of IL-12, which acts as the third signal in antigen presentation and co-stimulation required for successful T cell priming [27].

In this study, we show that rHDL interferes with the activation of human granulocytes, CD14^+^ monocytes and monocyte-derived DC (MoDC) at multiple levels by reducing immunostimulatory properties, secretion of proinflammatory cytokines and chemokines. Furthermore, rHDL inhibits TLR-induced activation of the transcription factor NF-κB.

## Materials and Methods

### Ethics statement

The volunteer blood donors signed an informed consent. In this document the donors are informed about the use of the donation. In addition, the donation is coded and transferred anonym to the research laboratory. The annual blood donation volume is defined and every second year the hemoglobin value is tested. There is a biannual screening for virus markers for HIV, HCV and HBV. The medical dossier is archived for 40 years. This internal donation system for research purposes is under the supervision of the medical services and was approved by an in-house ethical committee (CSL Behring) headed by the medical direction.

### Preparation of reconstituted High Density Lipoprotein (rHDL; CSL111)

rHDL (CSL111) was prepared as described in detail by Lerch *et al.* [28]. In brief, rHDL with a molar ratio of apoA-I to soybean phosphatidylcholine (PC) of 1:150 was prepared. Cholic acid sodium salt (3.08 kg) was dissolved in 25 liters of a buffer containing 10 mmol/l Tris-HCl, 10 mmol/l NaCl, 1 mmol/l EDTA, pH 8.0. In this buffer 4.2 kg PC were dissolved for 6 h at room temperature. The lipid solution was sterile-filtered (0.22 µm) and then mixed with 1 kg of apoA-I in 200 liters 10 mmol/l NaCl, and incubated for at least 2 h at 0–2°C. After the incubation the mixture was diafiltered with a Pellicon using Biomax cassettes (NMWR  = 10 kDa; Millipore) with at least 5 vol of a 1% sucrose solution. The protein concentration was then increased to approximately 2.5%, and the pH was adjusted to 7.5 with either 0.2 mol/l NaOH or 0.2 mol/l HCl. The protein concentration was determined by the Biuret method, sucrose was added to a final concentration of 10% and the concentration of the lipoprotein solution was adjusted to 2% protein concentration. After a final sterile filtration (0.22 µm) the rHDL was filled in bottles of 1 g rHDL (protein weight) and lyophilized.

### Stimulation and FACS analysis of leukocytes in whole blood

Heparinized whole blood from healthy volunteers was collected into pyrogen-free tubes, to which 5 μg/ml phytohemagglutinin-M (PHA, Calbiochem, Massachusetts, USA) was added for leukocyte stimulation. Simultaneously, rHDL was added to the whole blood at concentrations ranging from 0.04 to 1 mg/ml and incubated overnight at 37°C, 5% CO_2_ in a humidified atmosphere. Addition of substances resulted in a 1:2 dilution of human whole blood. The following day all manipulations were performed at 4°C or on ice. The cells were directly stained with antibodies specific for CD14, ICAM-1 and CD45 (BD Biosciences) for 30 min on ice. Red blood cells (RBC) were lysed by a 30 min incubation with EC Lysis Buffer (Qiagen AG, Basel, Switzerland) and gentle mixing. With the majority of RBC lysed, the tubes were centrifuged (400×g, 10 min, 4°C) and the cells resuspended in 300 μl PBS. Data acquisition and analysis was performed on a FACSCanto II flow cytometer employing the BD FACSDiva software (both BD Biosciences AG).

For analysis, leukocytes were identified by gating on the pan-leukocyte surface marker CD45 which enables to exclude remaining non-lysed RBC from analysis. Then granulocytes were separated using granularity (side scatter; SSC) and monocytes using CD14 as marker which is expressed on the majority of monocytes (>90% of the whole population). This procedure allowed the assessment of ICAM-1 expression on the cell surface of each of these cell populations (all antibodies from BD Biosciences). To compare the levels of up-regulation of the indicated surface molecules, the median fluorescence intensity (MFI) ratios were calculated by dividing the median fluorescence of PHA-treated gated cells populations i.e. granulocytes and CD14^+^ monocytes by the median fluorescence of non-treated cells (indicated as fold increase MFI).

### Measurement of cytokines and chemokines by Luminex multiplex array system

For the analysis of cytokine/chemokine production, supernatants were harvested after overnight stimulation as described above. The cytokine/chemokine levels in these supernatants were measured by using a commercial human cytokine magnetic 25-plex panel (Cat. LHC0009M, Invitrogen Life Technologies, Paisley, UK) according to manufacturer′s instructions. The panel consists of the following analytes: IL-1β, IL-1RA, IL-2, IL-2R, IL-4, IL-5, IL-6, IL-7, IL-10, IL-12p40, IL-13, IL-15, IL-17A, TNF-α, IFN-α, IFN-γ, GM-CSF, CCL-2 (MCP-1), CCL-3 (MIP-1α), CCL-4 (MIP-1β), CCL-5 (RANTES), CCL-11 (Eotaxin), CXCL-8 (IL-8), CXCL-9 (MIG), CXCL-10 (IP-10).

### Generation and stimulation of human monocyte-derived DC (MoDC)

Human peripheral blood mononuclear cells (PBMC) were isolated from buffy coats obtained from healthy blood donors (Regional Red Cross Blood Donation Center, Bern, Switzerland) by density gradient centrifugation over Ficoll-Paque (Amersham, Uppsala, Sweden). Monocytes were isolated from PBMC as described previously [29]–[31] by spontaneous aggregation and rosetting [32]. The purified monocytes were incubated for 6 days at a concentration of 10^6^ cells/ml in RPMI 1640 medium (Invitrogen Life Technologies) containing 10% fetal calf serum (FCS; Amimed/BioConcept), 1% [2mM] L-Glutamine (Invitrogen), 1% [100 U/ml] Penicillin/Streptomycin (Invitrogen), 10 ng/ml GM-CSF (R&D Systems Europe Ltd, Abingdon, Oxon, UK), and 10 ng/ml IL-4 (R&D) to generate MoDC as described initially by Sallusto and Lanzavecchia [33]. The cells were kept at 37°C in a 5% CO_2_ humidified atmosphere. On day 3, the culture medium was replaced with fresh medium. For induction of maturation 100 ng/ml LPS (Sigma), 5 μg/ml lipoteichoic acid (LTA, Sigma) or 20 μg/ml hyaluronic acid (HA, Sigma) were added to the cultures for the indicated time periods. Concentrations of the TLR agonists were chosen according previous published literature [31], [34], [35] . Treatment of HA with polymyxin B (Sigma), an inhibitor of LPS, did not affect biological activity of HA [34], indicating that the observed effects of HA were not due to potential LPS contamination. MoDC were pre-incubated with different concentrations of rHDL (0.016, 0.08, 0.04, 0.2, 1.0 mg/ml) for 30–60 minutes. After this period, maturation of MoDC was induced by LPS, HA or LTA. Cells were not washed before addition of TLR agonists.

### FACS analysis of MoDC and cell viability

Cells were incubated with FITC-labeled monoclonal antibody (mAb) against CD80, CD83 and CD86 (BD, Franklin Lakes, NJ, USA) or Isotype Control IgG1 (BD).

For determination of viability, propidium iodide (PI; Invitrogen; 5 μg/ml) was added to stained cells immediately before analysis by flow cytometry. As control for cell viability staining, cells were treated with PBS containing 0.1% BSA (Sigma) and 0.1% saponin (Sigma) and then stained with PI. To compare the levels of up-regulation of the indicated surface molecules, the median fluorescence intensity (MFI) ratios were calculated by dividing the median fluorescence of TLR-treated MoDC by the median fluorescence of non-treated MoDC (indicated as fold increase MFI). Measurements were performed with a BD FACScan flow cytometer and the obtained data were analyzed using FlowJo (Tree Star Inc., Ashland, OR, USA).

### Detection of NF-κB activation by a transcription factor ELISA

The production of NF-κB p65 was measured using an NF-κB assay kit (Active Motif, Rixensart, Belgium) according the manufacturer′s instructions. In brief, cell extract (10 μg of total protein, generated with the provided lysis buffer) of LTA activated DC, with or without additional pretreatment by rHDL (40 μg/ml) and untreated cells, was added to each well coated with consensus-binding site oligonucleotides of NF-κB p65. A primary antibody specific for an epitope on the bound and active form of the transcription factor was then added, followed by incubation with secondary HRP-conjugated antibody.

### Statistical analysis

Data are presented as mean ± standard deviation (SD) representing experiments with 3 to 5 different donors. Paired two tailed students *t-tests* were performed for evaluation of significance. Differences were considered statistically significant at p-values less than 0.05. Data were analyzed using GraphPad Prism software 5.04 (GraphPad, San Diego, CA).

## Results

### Secretion of proinflammatory cytokines is prevented by rHDL in whole blood

**Figure 1:**
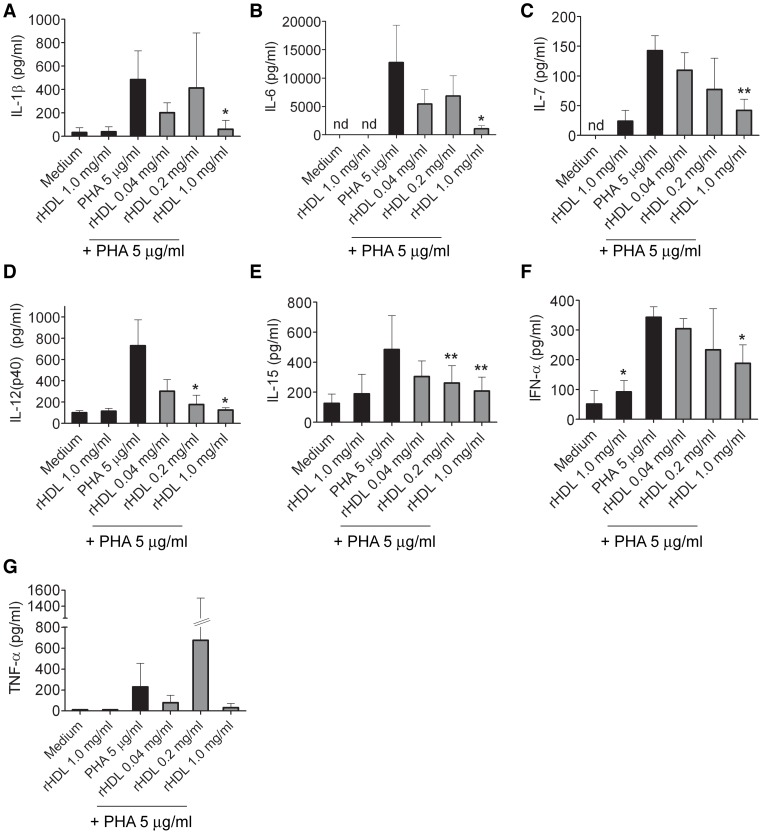
rHDL inhibits the PHA induced production of proinflammatory cytokines in human whole blood. Cytokines were measured by multiplex analysis in supernatants of overnight cultures for IL-1β (*A*), IL-6 (*B*), IL-7 (*C*), IL-12(p40) (*D*), IL-15 (*E*), IFN-α (*F*) and TNF-α (*G*). n.d.: not detectable. Mean values ± SD are shown as column graphs (n = 4). *p<0.05; **p<0.01 vs. PHA or medium (paired Student's *t*-test).

The biological effect of rHDL on the inhibition of an inflammatory reaction was first assessed in a human whole blood assay and screened by multiplex technology on production of various cytokines and chemokines. Over-night stimulation of whole blood with phytohemagglutinin (PHA; 5 µg/ml) led to a considerable secretion of IL-1β, IL-6, IL-12(p40), IL-15, TNF-α, and IFN-α (Fig. 1). No secretion of the pro-inflammatory cytokine IL-17A was detected (Lower limit of detection (LLOD): 51.15 pg/ml). Induction of IFN-γ by treatment with PHA was only detected in one donor (LLOD: 14.5 pg/ml) whereas GM-CSF was secreted in three donors out of four (LLOD: 23.98 pg/ml; data not shown). Co-incubation with rHDL reduced PHA-induced secretion of IL-1β, IL-6, IL-12(p40), IL-15 and IFN-α (Fig. 1). At an rHDL concentration of 1 mg/ml we observed significantly reduced secretion for all of these cytokines. No secretion of cytokines was induced by treating the cells with rHDL at 1 mg/ml in the absence of PHA, except for IFN-α, where a low, but significant increase was observed. No significant effect of rHDL on PHA-induced secretion of TNF-α was observed. There might have been a trend towards inhibition of TNF-α with 1 mg/ml rHDL. Surprisingly, there was a high variation in the TNF-α levels measured in PHA treated blood samples of the different donors when treated with 0.2 mg/ml of rHDL.

### Influence on secretion of chemokines by rHDL in whole blood

**Figure 2:**
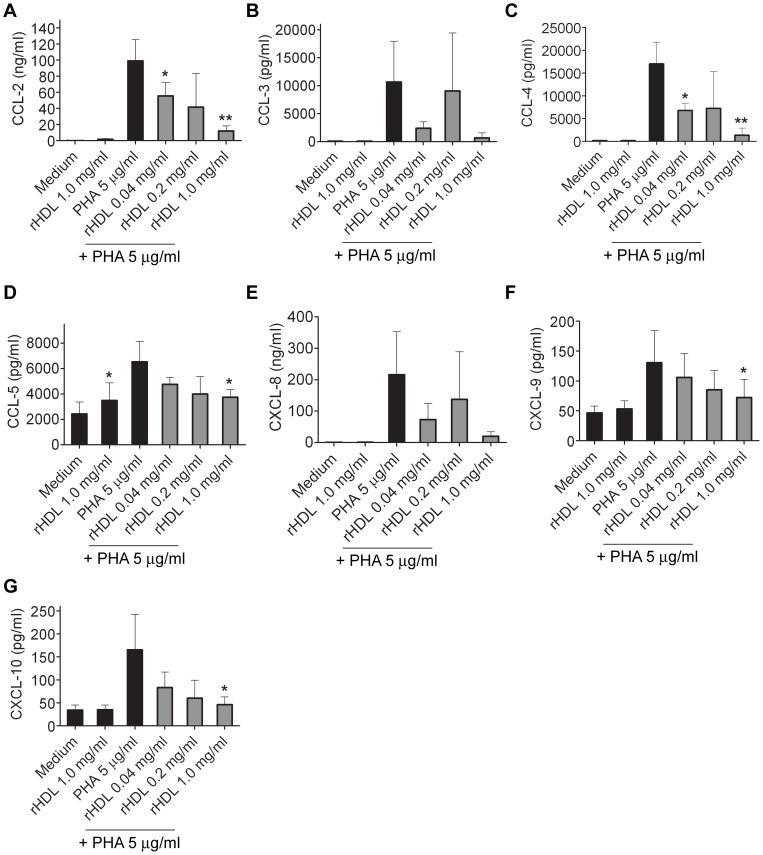
rHDL inhibits the production of proinflammatory chemokines in human whole blood. Chemokines were measured by multiplex analysis of overnight culture for CCL-2 (*A*), CCL-3 (*B*), CCL-4 (*C*), CCL-5 (*D*), CXCL-8 (*E*), CXCL-9 (*F*) and CXCL-10 (*G*). Mean values ± SD are shown as column graphs (n = 4). *p<0.05; **p<0.01 vs. PHA or medium (paired Student′s *t*-test).

PHA stimulation of whole blood led to a secretion of all measured chemokines. A significant inhibitory effect on the production of the chemokines CCL-2, CCL-4, CCL-5, CXCL-9 and CXCL-10 was observed at a concentration of 1 mg/ml of rHDL (Fig. 2). No significant effect of rHDL on the secretion of CCL-3, CCL-11 and CXCL-8 could be detected. However, there was trend towards inhibition of CCL-3 and CXCL-8 by 1 mg/ml of rHDL (Fig. 2).

### Influence on secretion of anti-inflammatory cytokines by rHDL in whole blood

**Figure 3:**
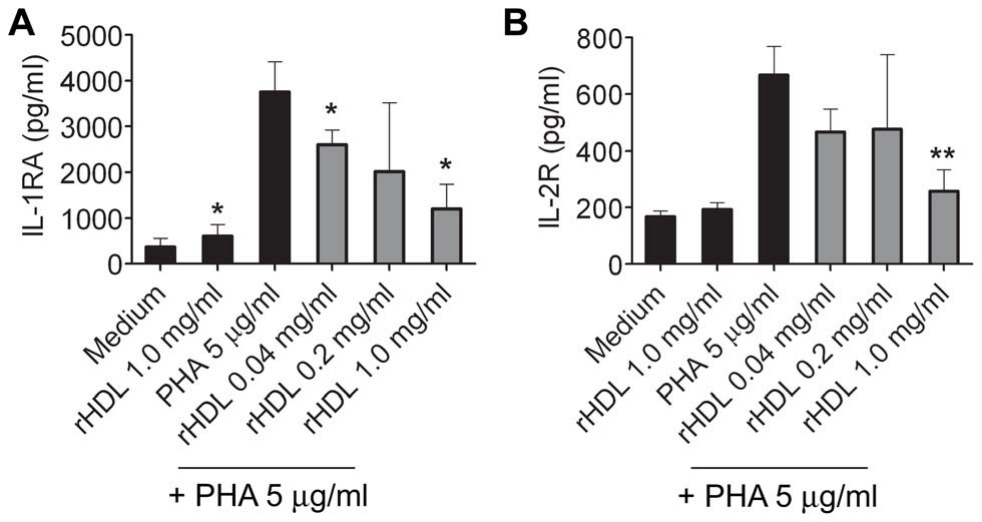
rHDL inhibits the production of anti-inflammatory cytokines in human whole human blood. Cytokines were measured by multiplex analysis of overnight culture for IL-1RA (*A*) and IL-2R (*B*). Mean values ± SD are shown as column graphs (n = 4). *p<0.05; **p<0.01 vs. PHA or medium (paired Student′s *t*-test).

The following cytokines that were measured in this study have been considered to possess anti-inflammatory properties: IL-1RA, IL-2R, IL-4, IL-5, IL-10 and IL-13. PHA induced secretion of IL-1RA and IL-2R was significantly reduced by rHDL (Fig. 3). No secretion of IL-4 and IL-5 was detected in the supernatants (LLOD IL-4: 58.49 pg/ml; LLOD IL-5: 3.27 pg/ml) of PHA activated cells. Secretion of IL-10 and IL-13 was detected in two out of four donors, all other values were below detection (LLOD IL-10: 30.1 pg/ml; LLOD IL-13: 28.15 pg/ml).

### Upregulation of ICAM-1 on CD14^+^ monocytes and granulocytes in whole blood is inhibited by rHDL in a dose-dependent manner

**Figure 4:**
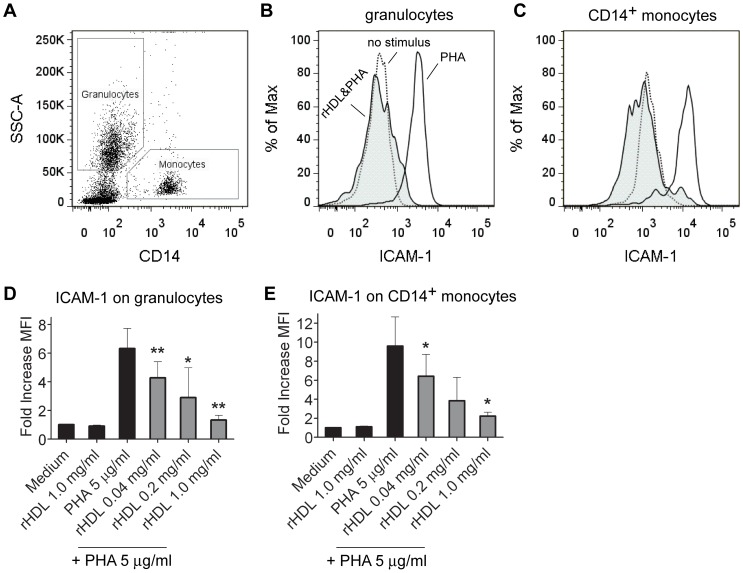
Stimulation of neutrophils and monocytes contained in human whole blood by PHA is dose-dependently inhibited by coincubation with rHDL. Surface expression of ICAM-1 (CD54) was measured on primary human granulocytes and CD14^+^ monocytes after overnight culture. *A,* Red blood cells (RBC) were lyzed prior the FACS analysis and remaining RBC were excluded from analysis by the leukocyte marker CD45. The shown representative dot-blot for CD14/SSC represents only CD45^+^ cells. Granulocytes were identified according the granularity (side scatter; SSC) and monocytes by CD14 expression. Cellular activation was evaluated by the upregulation of ICAM-1 on the respective cellular subset. *B* and *C*, Histograms show the typical expression profiles of ICAM-1 on neutrophils (*B*) and CD14^+^ monocytes (*C*). Data are representative of 4 independent experiments with cells of different donors. Grey histograms show the typical expression profile of ICAM-1 for rHDL (1 mg/ml) plus PHA (5 µg/ml) treated cells. White: PHA only treated cells; Dotted line: no stimulus. *D* and *E*, To compare the levels of up-regulation of the indicated surface molecules, the median fluorescence intensity (MFI) ratios were calculated by dividing the median fluorescence of PHA- and/or rHDL-treated cells by the median fluorescence of untreated cells (medium control) and indicated as fold increase in the MFI. Mean values ± SD are shown as column graphs (n = 4). *p<0.05; **p<0.01 vs. PHA (paired Student′s *t*-test).

Many of the cytokines the excretion of which was affected by rHDL are produced by cells of myeloid origin, namely granulocytes, monocytes, macrophages or DC. Therefore, we analyzed the expression of ICAM-1 (CD54) as a marker of cellular activation [36] on CD14^+^ monocytes and granulocytes (Fig. 4*A*). Incubation with rHDL dose-dependently inhibited the up-regulation of ICAM-1 on human granulocytes (Fig 4*B and D*) and CD14^+^ monocytes (Fig. 4*C and E*).

### Phenotypic maturation induced by endogenous as well as exogenous TLR4 agonists of human MoDC is prevented by rHDL

**Figure 5:**
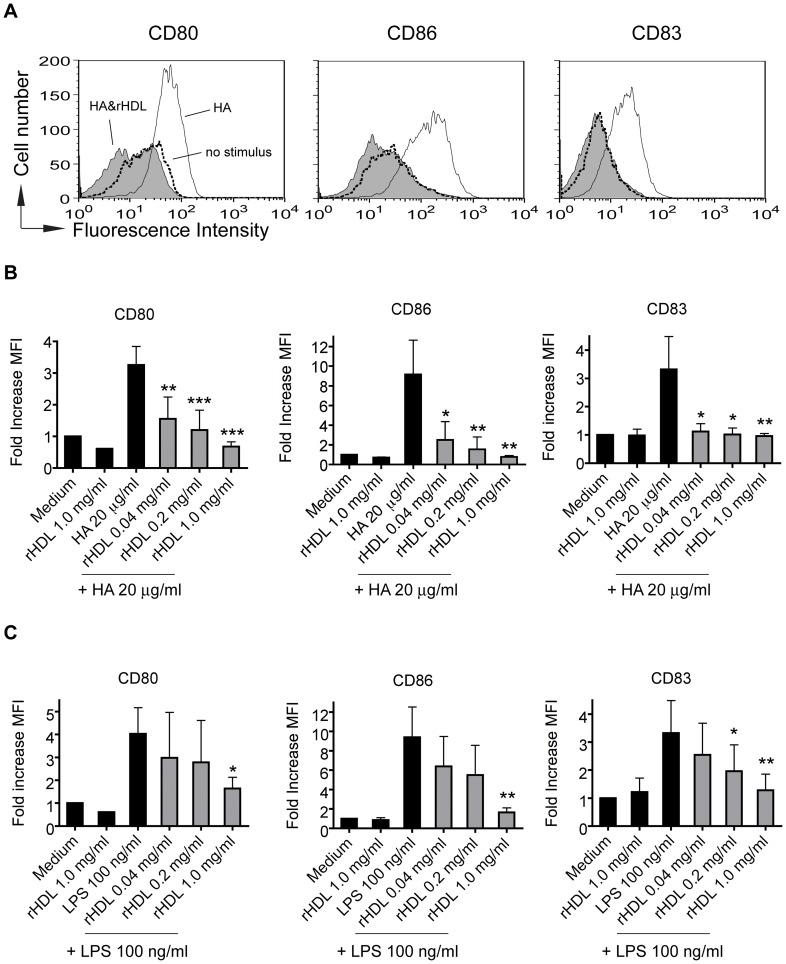
rHDL prevents phenotypic maturation of human MoDC in response to HA (20 μg/ml) and LPS (100 ng/ml) in a dose dependent manner. *A*, Monocytes isolated from buffy coat were cultured in presence of GM-CSF and IL-4 for 6 days. rHDL dose-dependently prevents HA-induced MoDC maturation. Histograms show the typical expression profiles of CD80, CD86 and CD83. Data are representative of 5 independent experiments with cells of different donors. Grey histograms show the typical expression profiles of the indicated surface molecules for rHDL plus HA treated MoDC. White: HA only treated cells; Dotted line: no stimulus. *B*, To compare the levels of up-regulation of the indicated surface molecules, the median fluorescence intensity (MFI) ratios were calculated by dividing the median fluorescence of HA- and/or rHDL-treated MoDC by the median fluorescence of immature MoDC and indicated as fold increase in the MFI. Mean values ± SD are shown as column graphs (n = 5). *p<0.05; **p<0.01; ***p<0.001 vs. mature MoDC (unpaired Student′s *t*-test). *C*, LPS- and/or rHDL-treated MoDC. Mean values ± SD are shown as column graphs (n = 5). *p<0.05; **p<0.01 vs. mature MoDC (unpaired Student′s *t*-test).

The frequency of myeloid DC in the peripheral human blood is very low (about 0.5% of all mononuclear cells) [37]. To investigate the effect of rHDL on DC maturation we worked therefore with monocyte-derived DC. As PHA also acts as a polyclonal stimulus on lymphocytes, we have used TLR agonists to more specifically activate myeloid cells. It has been described that LPS derived from *E. coli* leads to maturation of human MoDC via TLR4, after the formation of a TLR4-signaling complex containing MD-2, CD14 and TLR4. HA has been described as endogenous TLR4 agonist, inducing the formation of a unique TLR4 complex consisting of MD-2, CD44 and TLR4 [38]. MoDC were pre-incubated with different concentrations of rHDL for 30 minutes, followed by induction of maturation with LPS or HA. As shown in Fig. 5, rHDL dose-dependently inhibited TLR4-induced phenotypic MoDC maturation measured by the upregulation of CD80, CD83 and CD86. A significant inhibitory effect of rHDL on HA-induced maturation was already observed at an rHDL concentration of 40 μg/ml whereas for LPS-induced activation a significant inhibition was achieved only at higher concentrations i.e. 0.2 mg/ml (for CD83) or 1.0 mg/ml (for CD80 and CD86).

### rHDL has an inhibitory effect on TLR2 induced maturation of human MoDC

**Figure 6:**
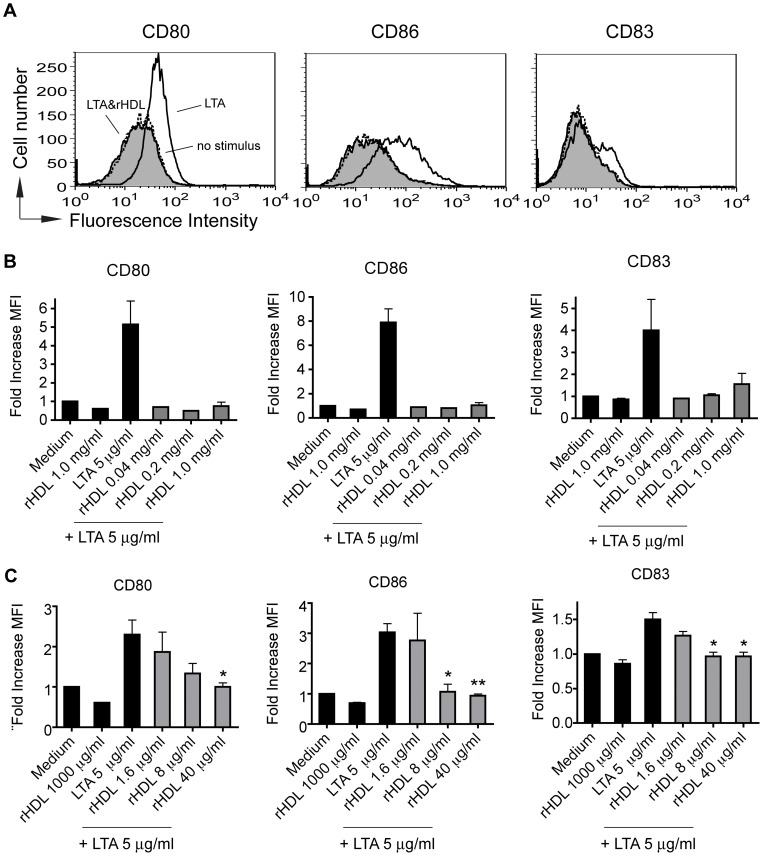
rHDL prevents phenotypic maturation of human MoDC in response to LTA in a dose dependent manner. *A*, rHDL dose-dependently prevents LTA-induced (5 μg/ml) MoDC maturation. Histograms show the typical expression profiles of CD80, CD86 and CD83. Grey histograms show the typical expression profiles of the indicated surface molecules for rHDL plus LTA treated MoDC. White: LTA only treated cells; Dotted line: no stimulus. *B* and *C*, To compare the levels of up-regulation of the indicated surface molecules, the median fluorescence intensity (MFI) ratios were calculated by dividing the median fluorescence of LTA- and/or rHDL-treated MoDC by the median fluorescence of immature MoDC and indicated as fold increase in the MFI. Mean values ± SD are shown as column graphs (n = 2 for *B*, n = 3 for *C)*. *p<0.05; **p<0.01 vs. mature MoDC (unpaired Student′s *t*-test).

LTA from gram-positive cocci *S. aureus* has been demonstrated to induce signaling through TLR2 [39]. LTA-activated MoDC were incubated with the same concentrations of rHDL (0.04, 0.2 and 1.0 mg/ml) as for TLR4 stimulation. Even at 40 μg/ml we could observe an almost complete inhibition of maturation (Fig 6*B*). As a consequence of this observation, we used lower concentrations of rHDL (1.6, 8.0 and 40 μg/ml) for subsequent experiments. As shown in Fig. 6*A and C*, at concentration of 8 μg/ml we could observe a significant inhibitory effect of rHDL on LTA induced up-regulation of CD83 and CD86 on human MoDC.

### Activation of NF-κB in response to LTA is prevented by rHDL

**Figure 7:**
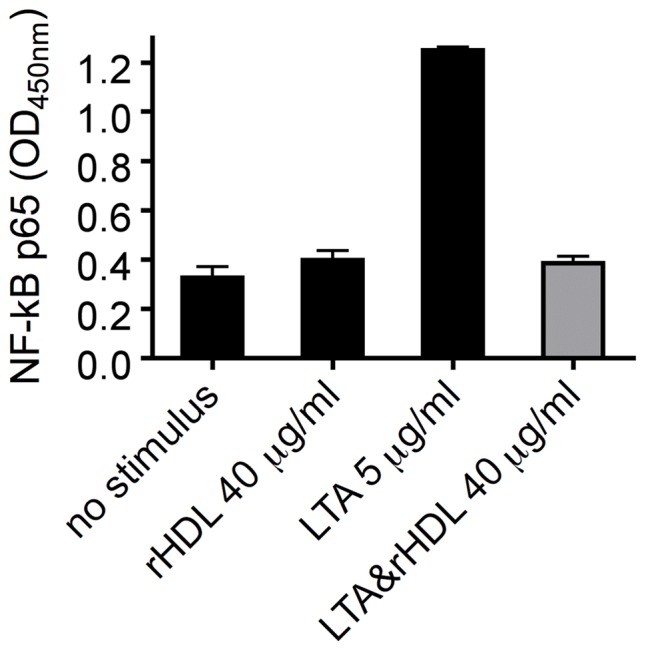
LTA induced activation of NF-κB is inhibited by rHDL. NF-κB activation was measured in cell extracts at 1 hour after LTA induced activation by a transcription factor ELISA. Cells were preincubated 30 min before LTA stimulation with rHDL (40****μg/ml). A representative experiment performed in duplicates from one donor out of three is shown. The bars represent mean ± SD.

The phosphorylation of IκB-α leads to its ubiquitylation and subsequent degradation, which results in a release of NF-κB, the essential transcription factor for DC maturation and function [40]. As shown by transcription factor ELISA, treatment of MoDC with LTA for one hour led to activation and translocation of NF-κB p65 into the nucleus, whereas pretreatment of the cells with rHDL caused a strong abrogation of this LTA-induced activation of NF-κB (Fig. 7).

### rHDL does not affect viability of human MoDC

To investigate putative effects of rHDL on the viability of MoDC, cell survival after a 24 hours incubation with increasing rHDL concentrations was monitored by using PI staining followed by immediate FACS analysis. Up to the highest rHDL concentration tested (1 mg/ml), either alone or in combination with LPS, LTA or HA, no effect on viability of MoDC was found (data not shown).

### Kinetic analysis of the effect of rHDL on the up-regulation of CD86

**Figure 8:**
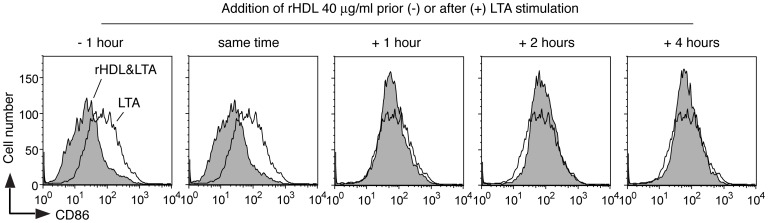
Effect of rHDL on MoDC maturation given prior to or after TLR stimulation. rHDL (40 μg/ml) was given to the cells at the indicated time point prior to or after stimulation with LTA (5 μg/ml) for 24 hours. The cells were then evaluated for the expression of CD86 by flow cytometry. The results shown are from one donor and representative of two independent experiments with cells from different donors.

To assess the kinetics of rHDL-mediated inhibition of MoDC maturation, cells were incubated with rHDL at different time periods prior to or after stimulation with LTA. As shown in Fig. 8, only pretreatment of the cells or co-stimulation with rHDL was able to prevent up-regulation of CD86.

## Discussion

In the present study we have demonstrated that rHDL, a substance with known beneficial clinical activity on coronary atherosclerosis (ERASE study; [1]), attenuated PHA-induced secretion of various cytokines and chemokines, in a human whole blood assay. The cellular sources of these pro- and anti-inflammatory mediators are primarily myeloid progenitors of the innate immune system. Subsequently, we have demonstrated an rHDL-mediated inhibition of activation of CD14^+^ monocytes and granulocytes. Furthermore, rHDL significantly inhibited up-regulation of the essential co-stimulatory molecules on human myeloid DC.

Earlier studies demonstrated an inhibitory effect of rHDL on LPS induced secretion of TNF-α, IL-1RA, IL-6, IL-10 or CXCL-8 in humans volunteers [41]. Furthermore, reconstituted HDL shown to significantly inhibit CCL-2 production in a periarterial collar model of blood vessel occlusion in normocholesterolemic rabbits in-vivo [42]. Furthermore, expression and secretion of CCL-2, CCL-5 and CX_3_CL-1 by human coronary artery endothelial cells as well as monocytes was inhibited by preincubation with rHDL [43], and rHDL (CSL111; 80 mg/kg) infused in patients with peripheral vascular disease decreased CD11b on neutrophils [44]. In addition, native HDL inhibits the secretion of IFN-γ and IL-12(p40) secretion by human MoDC [45] and CCL-2 production by rat vascular smooth muscle cells [46]. Several studies investigated whether the observed anti-inflammatory effect of HDL is mediated via apoA-I or the phospholipids. Hyka *et al.* demonstrated an inhibitory effect of apoA-I and delipidated HDL on production of TNF-α and IL-1β by activated monocytes and they observed an inhibitory effect of apoA-I on secretion of TNF-α and IL-1β by PHA-stimulated PBMC [47]. Moreover, it has been shown that apoA-I modulates differentiation of human monocytes into DC *in-vitro* [48]. A recent study explored the effect of high-density lipoprotein phospholipids on DC maturation and their capacity to induce T cell activation. An inhibitory effect of high-density lipoprotein phospholipids on LPS mediated secretion of IL-12(p40) by MoDC was observed and DC mediated production of IFN-γ by T cells was significantly reduced [49]. Overall, the anti-inflammatory properties of HDL or rHDL may not exclusively be mediated by the protein or the lipid compound and we therefore investigated the anti-inflammatory properties of the whole rHDL particle.

As an initial step to investigate the anti-inflammatory properties of rHDL we analyzed the influence of rHDL on cytokine and chemokine secretion in human whole blood after PHA stimulation by a multiplex assay. Although PHA is not a physiological activator of human immune cells, it is a potent and robust activator of leukocytes in whole blood. PHA was therefore considered a valuable stimulus to investigate the effect of rHDL on secretion of cytokines and chemokines in a human whole blood system. We demonstrate a novel inhibitory effect of rHDL on the secretion of IL-1β, IL-2R, IL-7, IL-12(p40), IL-15, IFN-α as well as the chemokines CCL-2, CCL-4, CCL-5, CXCL-9 and CXCL-10. Many of these proinflammatory cytokines and chemokines are involved in the pathology of various human diseases such as atherosclerosis [50], [51], ACS [17] or I/R injury [52]–[54]. Moreover, rHDL had a dose-dependent inhibitory effect on PHA-induced production of IL-12(p40). The heterodimeric proinflammatory cytokine IL-12 is known to be a potent inducer of IFN-γ secretion [55]. Importantly, IL-12 has been regarded as a third signal besides antigen presentation and co-stimulation required for successful T cell priming [27].

Several known immunomodulating agents have been demonstrated to upregulate the production of anti-inflammatory cytokines, as mainly demonstrated for IL-10. For example intravenous immunoglobulins (IVIG) have been shown to increase the secretion of IL-10 by DC [56] or whole blood [57]. DC were incubated with IVIG for 12 hours before LPS was added as maturation stimulus for additional 48 hours [56]. Longer preincubation or incubation periods of the cells with rHDL may therefore be necessary prior to activation with PHA or TLR agonists to induce enhanced secretion of IL-10. Other substances as e.g. glucocorticoids have been shown to increase secretion of IL-10 in DC [58], whereas in LPS-stimulated human whole blood the effect was biphasic, i.e. induction at low doses and inhibition at higher doses [59]. In our hands, however, in the whole blood assay no increased secretion of any cytokine with potential anti-inflammatory properties such as IL-1RA, IL-2R, IL-4, IL-5, IL-10 or IL-13 was found after incubation of the cells with rHDL. Induction, regulation and kinetics of these cytokines is complex and depends on the cell type, stimulus and concentration of the used agonist. IL-10 is a well described and studied anti-inflammatory cytokine, which is mainly produced various as e.g. macrophages after stimulation with microbial products mainly controlled by the transcription factor NF-κB. Beside microbial stimuli, additional signals might be required for IL-10 production, as e.g. type I interferons. One could speculate whether the observed inhibition of proinflammatory cytokines by rHDL prevents a subsequent increase or induction of anti-inflammatory cytokines [60].

On a cellular level, we demonstrated a dose-dependent inhibition by rHDL of the PHA-induced up-regulation of ICAM-1 on the surface of CD14^+^ monocytes and granulocytes. Expression of ICAM-1 is necessary on monocytes for interaction with LFA-1 (CD11a/CD18), expressed on T lymphocytes and therefore crucial for successful T cell priming during antigen presentation and subsequent generation of an adaptive immune response [61]. As rHDL inhibited PHA-induced activation of CD14^+^ monocytes, the possible influence of rHDL on maturation of human MoDC was examined. Endogenous agonists of TLR have been demonstrated to trigger sterile inflammatory responses such as I/R injury, graft rejection, atherosclerosis or ACS [62]. Our data show an inhibitory effect of rHDL for TLR4- (HA, LPS) as well as TLR2-mediated stimulation (LTA). As rHDL has been reported to bind and neutralize LPS [41], the effect on LPS induced maturation of MoDC was expected. However, we observed a much more potent inhibitory effect of rHDL on HA or LTA-induced maturation. A concentration of 40 μg/ml was sufficient to significantly prevent NF-κB activation and subsequent maturation of MoDC. Modulation of DC by e.g. maintaining them in an immature or semimature state has been suggested to favor induction of immunological tolerance [63], [64]. Liver X receptors (LXRα and LXRβ) are oxysterol-activated transcription factors which sense elevated cellular cholesterol levels and trigger, when activated, a transcriptional program for cholesterol efflux as e.g. the ATP-binding cassette transporter (ABC) A1 or G1 (ABCA1, ABCG1). Interestingly, LXR deficient mice develop age-dependent systemic autoimmune disease with autoantibody production and autoimmune glomerulonephritis. Macrophages, immature as well as mature DC activated with a LXR agonist increased phagocytic activity of apoptotic thymocytes and exhibited a tolerogenic signature by e.g. secreting higher levels of IL-10 and TGF-β. Macrophages or DC deficient for LXR exhibited a defect in phagocytosis and maintain secretion proinflammatory cytokines as e.g. IL-1β [65].

Earlier mechanistic studies implicate cholesterol efflux via ABCA1 or ABCG1 from cellular membranes and subsequent modulation of cellular activity and function of the innate and adaptive immune system [66]. Furthermore, ABCA1 expression has been suggested to dampen MyD88-dependent TLR signaling and myeloid-cell specific ABCA1 knock-out mice have been demonstrated to be less susceptible to *L. monocytogenes* infection [67]. ABC ligation is thought to modify lipid raft abundance, potentially constituting the main mechanism by which rHDL inhibits cell activation [9], [68], [69]. A recent study demonstrate that rHDL (CSL111) induces a time- and dose-dependent increase of cholesterol efflux via ABCA1 and scavenger receptor type BI (SR-BI) *ex-vivo* [70]. Whether inhibition of PHA-induced cytokine and chemokine responses in whole blood or TLR-mediated maturation of MoDC by rHDL acts in a similar way, needs to be evaluated. rHDL does not directly bind PHA (Diditchenko, S. *et al.,* accepted for publication). However, it is yet unclear whether rHDL binds and neutralizes the TLR agonists HA or LTA and further studies are warranted to answer this question. Nevertheless, several studies have demonstrated that HA can act as a danger signal in sterile inflammation as recently reviewed and summarized by Chen *et al.* [71]. LTA and peptidoglycan from *S. aureus* have been suggested to act as important molecules to cause gram positive septic shock and multiple organ failure [72]. It has been demonstrated that HDL could inactive the LTA mediated activation of the murine macrophage cell line RAW264.7 in the presence of lipopolysaccharide-binding protein [73]. Binding and neutralization of HA or LTA in inflammatory conditions might be of therapeutic value.

It should be noted that the rHDL concentrations used do not affect viability and metabolic activity of the cells.

In conclusion, our data suggest a new immunomodulatory function and anti-inflammatory effect of rHDL primarily by inhibition of pro-inflammatory cytokine induction. Besides the obvious role in lipid metabolism this compound might therefore be useful as a therapeutic agent to impede early cellular inflammation and to impact on the link between innate and adaptive immunity.
